# Formation of epoxide-amine oligo-adducts as OH-functionalized initiators for the ring-opening polymerization of *ε*-caprolactone

**DOI:** 10.3762/bjoc.6.105

**Published:** 2010-10-01

**Authors:** Julia Theis, Helmut Ritter

**Affiliations:** 1 Institut für Organische Chemie und Makromolekulare Chemie, Heinrich-Heine-Universität Düsseldorf, Universitätsstraße 1, 40225 Düsseldorf, Germany

**Keywords:** addition oligomerization, epoxide-amine adducts, microwave, ring-opening polymerization, transfer hydrogenation

## Abstract

Epoxide-amine oligo-adducts were synthesized via a one-pot microwave assisted heterogeneous catalytic transfer hydrogenation. Accordingly, 4-nitroanisole was reduced under microwave conditions to give 4-aminoanisole which reacted immediately with the diglycidyl ether of bisphenol A in an addition polymerization reaction to yield oligo(amino alcohol)s. The hydroxy groups of the new formed oligomers were used as the initiator for the ring-opening polymerization of *ε*-caprolactone to produce a graft copolymer.

## Introduction

In the last decade the use of microwave (MW) irradiation in organic, pharmaceutical and polymer chemistry has become a well-established technique to promote chemical reactions. In many cases, the main advantages of this physical heating method over traditional heating in an oil bath are increases in reaction speed, product yields and purity [1–10].

Some previous works discussed the effective experimental procedure of metal-catalyzed reactions inside a MW oven [11–12]. Among the several types of these catalytic reactions, hydrogenation is probably one of the most useful in synthetic chemistry. Since many laboratory MW reactors are constructed to work under pressure, the MW tube can be regarded as a small autoclave. Therefore, this reaction can be performed either by using gaseous hydrogen [13–14] or by addition of a hydrogen source such as ammonium formate [15–19] or methylcyclohexenes [20].

Furthermore, the addition polymerization of primary amines with diepoxides forming linear adducts has been intensively studied [21]. Linear epoxide-amine addition polymers can be obtained for instance by the addition reaction of diglycidyl ethers and aromatic primary amines in equimolar amounts. With regard to our former work on the formation of hyperbranched epoxide-amine adducts via microwave-assisted heterogeneous catalytic transfer hydrogenation [22], we report herein the synthesis of an linear oligo(amino alcohol). The hydroxy groups of the latter were then used as an initiator for the ring-opening polymerization (ROP) of *ε*-caprolactone to construct a new class of graft copolymers.

## Results and Discussion

### Formation of epoxide-amine adducts

**Scheme 1:**
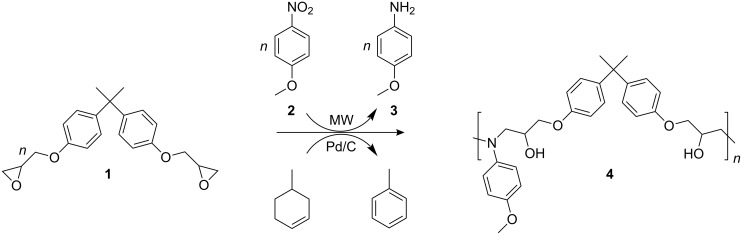
One-pot synthesis of epoxide-amine adducts via MW-assisted transfer hydrogenation.

Epoxide-amine adducts based on diglycidyl ether of bisphenol A (**1**) and 4-aminoanisole (**3**) were synthesized in a MW-assisted one-pot reaction. Thus, 4-nitroanisole (**2**) was reduced to the corresponding amine **3** via catalytic transfer hydrogenation. In presence of **1**, a simultaneous addition reaction of the freshly formed amine groups took place (Scheme 1). The reaction was carried out using 4-methyl-1-cyclohexene as a source of hydrogen in bulk. To prevent overheating, the reaction was carried out under simultaneous cooling with 5 psi of compressed air.

The obtained epoxide-amine addition product **4** was characterized by ^13^C NMR, Fourier transform infrared (FT-IR) spectroscopy and matrix-assisted laser desorption-ionization time-of-flight (MALDI-TOF) mass spectrometry. The successful reduction of the nitroaromatic compound **2** was proven by IR spectroscopy by the disappearance of the asymmetric and symmetric NO_2_ stretching vibration absorption bands at 1589 and 1329 cm^−1^, respectively. The addition reaction of the new formed primary aromatic amine **3** with diepoxide **1** was proven by the complete disappearance of the absorption band at 911 cm^−1^ associated with the C–H stretching vibration of the epoxide group. Furthermore, the formation of amino alcohol units was confirmed by the appearance of a broad band in the range of 3600–3150 cm^−1^ with a maximum at 3370 cm^−1^ corresponding to the O–H stretching vibration. The absence of both epoxide and N–H-amine absorption bands indicates the formation of some cyclic compounds [23].

**Figure 1:**
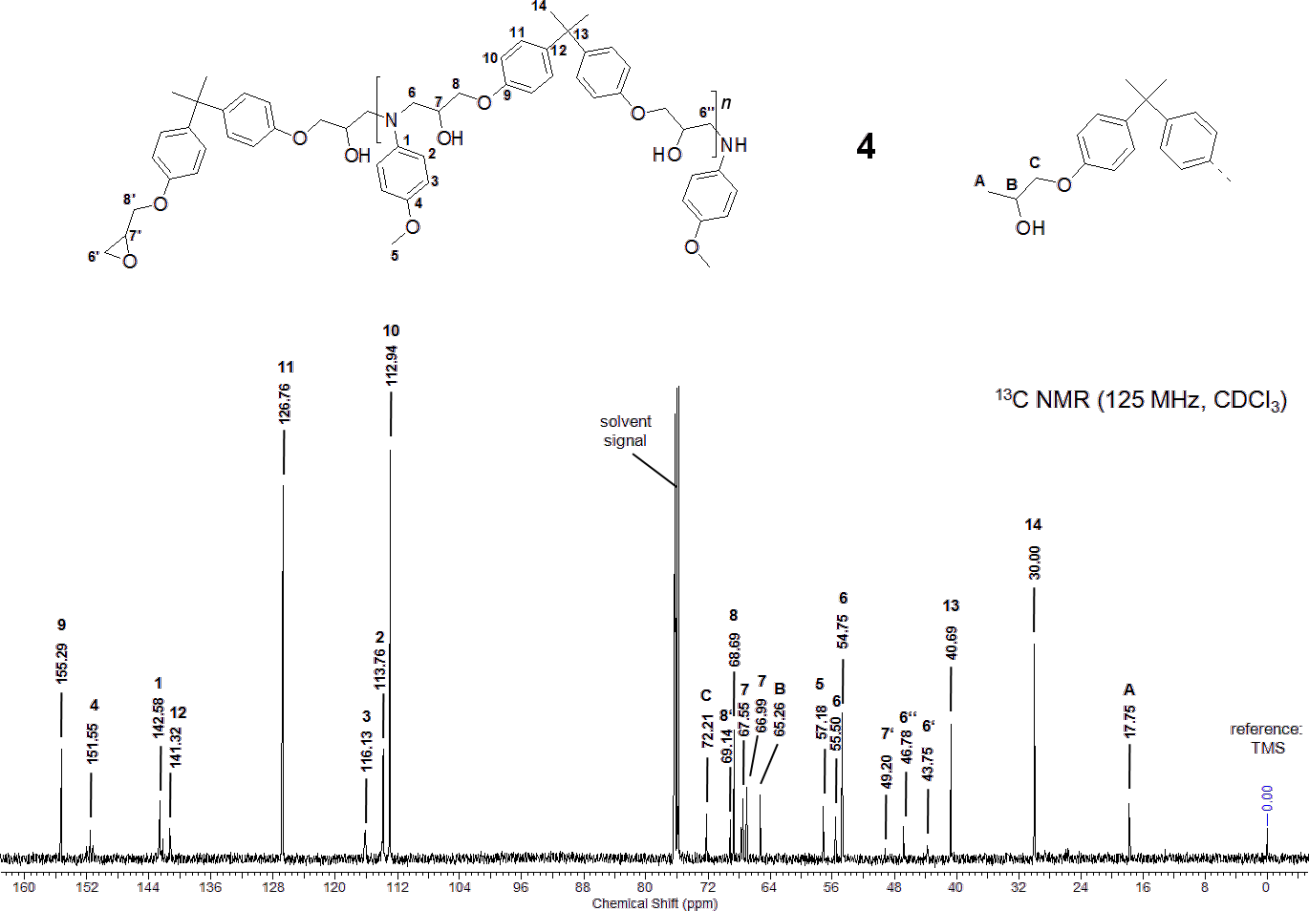
^13^C NMR spectra of **4**, measured in CDCl_3_.

The ^13^C NMR spectra of **4** (Figure 1) was compared with the data given in Ref. [24]. All characteristic signals of the formed addition product **4** were observed. Two signals for C-atom 6 appear due to the diastereomeric erythro and threo amino-diol units. Additionally, two signals for C-atom 7 were observed. The signals of the epoxide groups which should appear at about 44 and 50 ppm were faintly visible. In addition, new signals at about 18, 65 and 72 ppm appeared.

It is known, that epoxide-compounds can be reduced under palladium catalysis to form alcohols [25–26]. The new signals at 18, 65 and 72 ppm suggest the formation of a secondary alcohol formed by the reduction of the epoxide end groups under the reaction conditions.

The presence of some epoxide groups was also established by ^1^H NMR spectroscopy. Because of overlap in the region between 3 and 4.5 ppm and the highly complex splitting of signals, ^1^H NMR spectra were of little use for an analysis of this kind of polymer. However, the existence of intact epoxide groups was proven by means of two very weak signals, which were difficult to discern, at 2.81 and 2.66 ppm, corresponding to the CH_2_ of the epoxide group.

**Figure 2:**
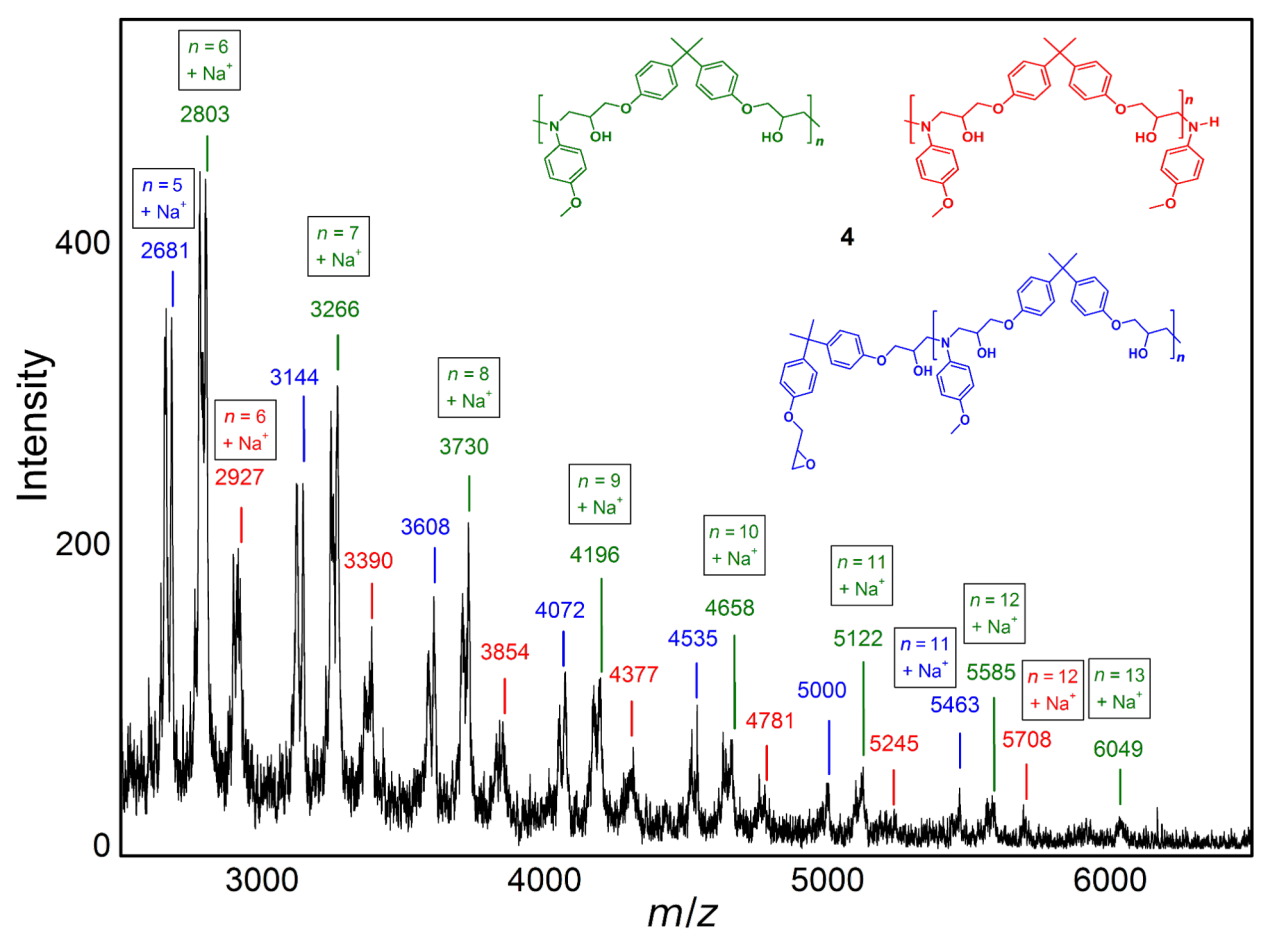
MALDI-TOF MS spectra (linear mode) of epoxide-amine product **4**.

MALDI-TOF MS measurements (Figure 2) definitely indicated the formation of an oligomer homologous series of epoxide-amine addition products of **1** and **3**. The amino alcohol repetitive unit (green) has a molecular mass of 463.56 g/mol and the molecule with the highest assignable molecular mass is the *n* = 13 oligomer with [M + Na^+^] = 6049 *m/z*. Furthermore, the mass to charge ratios (*m/z*) of the repetitive unit containing epoxide (blue) and amine (red) end groups were observed.

**Figure 3:**
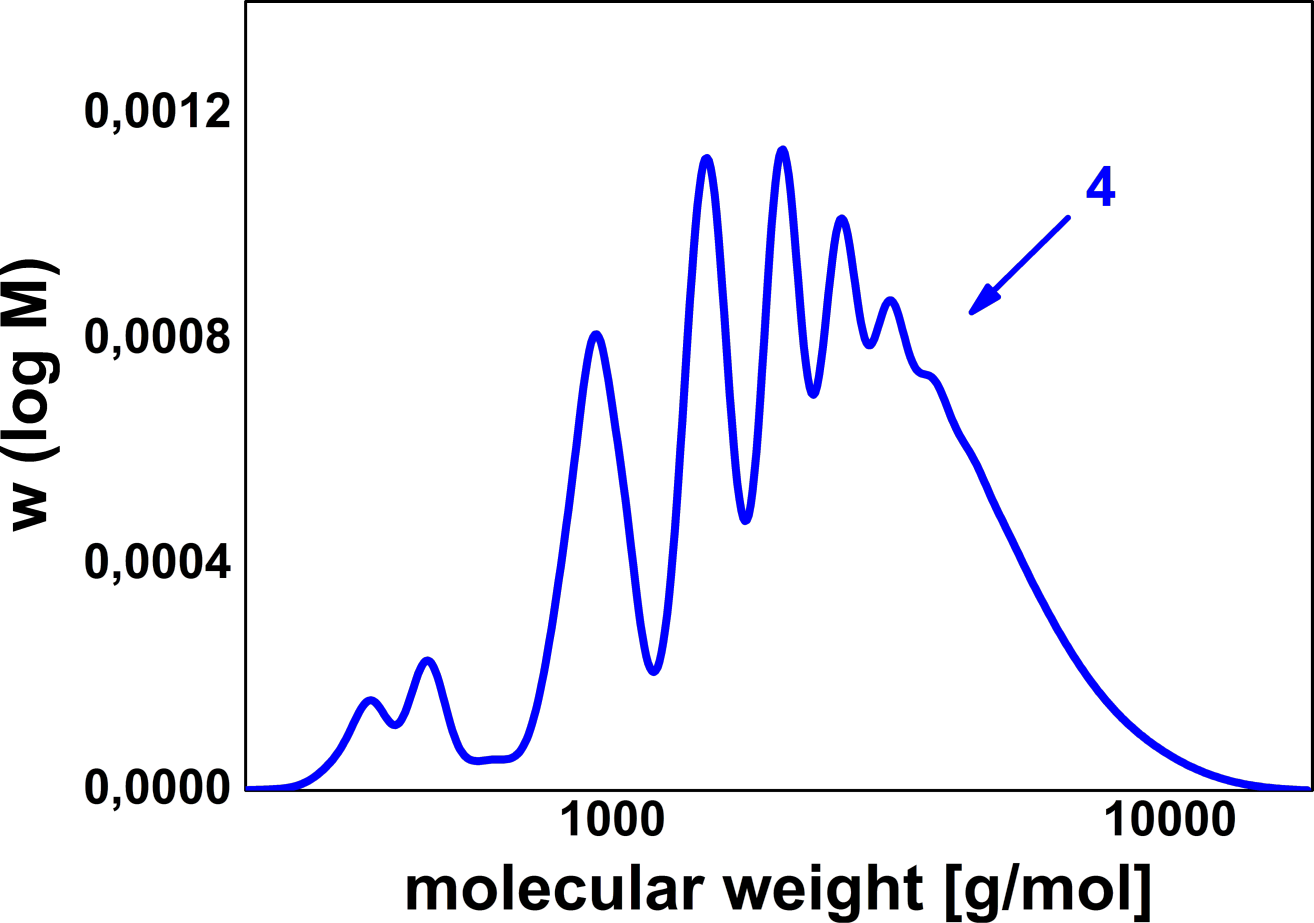
GPC curve of epoxide-amine product **4** detected by UV absorption.

The presence of oligomeric compounds was also confirmed by dynamic light scattering (DLS) measurements in dimethylformamide which indicated a number-average hydrodynamic diameter of 1.45 nm. Moreover, the gel permeation chromatography (GPC) diagram (Figure 3) clearly illustrates the formation of oligomers with repetitive units.

Molecular weights calculated from the GPC curve are about 1700 g mol^−1^ for *M*_n_ and 2600 g mol^−1^ for *M*_w_. The molecular weight distribution of the addition polymer **4** is relatively narrow (*M*_w_/*M*_n_ = 1.6) which is typical for low molecular weight polymers.

The differential scanning calorimetry (DSC) curve of compound **4** shows a glass transition temperature (*T*_g_) at 63 °C. The low molecular weight epoxide-amine adduct **4** is soluble, e.g., in tetrahydrofuran (THF), methanol, chloroform but insoluble in *n*-hexane and water.

**Figure 4:**
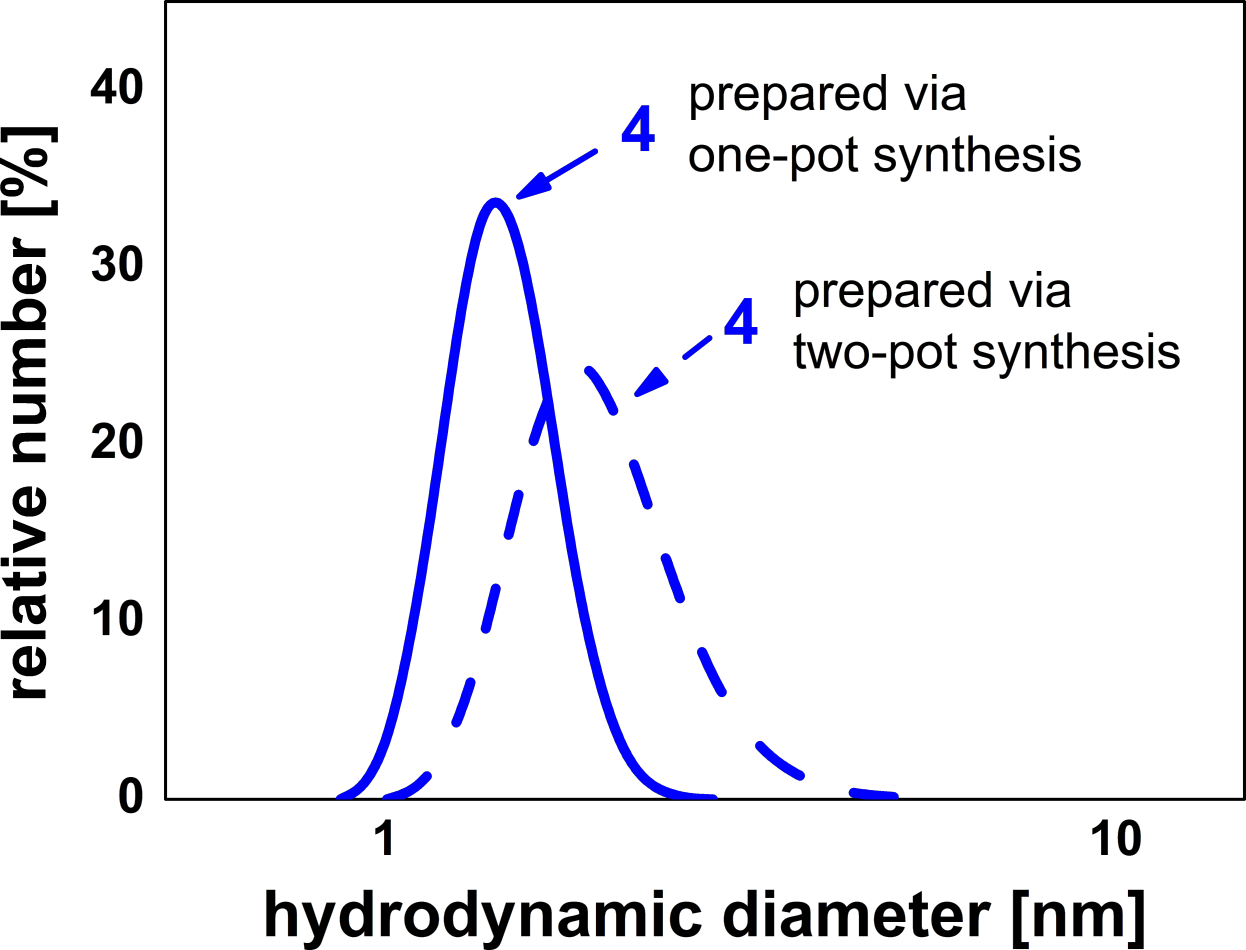
Number-average hydrodynamic diameters of compound **4** prepared via the one-pot (continuous line) and the two-pot (dashed line) synthesis.

We also carried out the above described one-pot reaction in THF as solvent. Surprisingly, in this process only reduction of the nitro groups took place, but no formation of oligomers was observed. Performing the solvent-free one-pot synthesis with reaction times up to 1 h did not lead to higher molecular weights. The formation of low molecular weight adducts **4** here is probably a result of the hydrogenation of some of the epoxide groups. Due to this side reaction, epoxide and amine compounds are not present in equimolar amounts so that the formation of higher sequences is prevented. To investigate this point, the addition polymerization was also carried out as a two-pot synthesis in bulk. Thus in the first step nitro compound **2** was reduced to the amine **3** according to Ref. [20]. Then, the crude product **3** was allowed to react with an equimolar amount of **1** for 10 min at 120 °C in the MW oven. As expected, the *M*_n_ and *M*_w_ values of the resulting product are about 4300 and 9300 g mol^−1^, respectively which are clearly higher than the values achieved in the one-pot synthesis as described above. The DSC curve of the addition product **4** synthesized via two-pot reaction shows a *T*_g_ at 76 °C which is an increase of 7 °C compared with the product obtained via the one-pot synthesis (*T*_g_: 63 °C). Figure 4 shows the number-average hydrodynamic diameters of addition product **4** prepared via the one-pot synthesis (continuous line) and via the two-pot synthesis (dashed line). Consistent with the GPC findings noted above, the number-average hydrodynamic diameter increases from 1.45 nm (one-pot) to 2.00 nm (two-pot).

Both FT-IR and ^13^C NMR spectra clearly show signals of epoxide end groups so that probably linear epoxide-amine adducts were formed under the two-step reaction conditions. In addition, the ^13^C NMR signals at about 18, 65 and 72 ppm which suggest the formation of a secondary alcohol formed by reduction of the epoxide end groups were absent in the ^13^C NMR spectra of **4** synthesized in the two-pot reaction. These findings indicate that in case of the one-pot synthesis the epoxide reduction to alcohols may take place as a significant side reaction which prevents the formation of higher molecular weight products.

### Ring-opening polymerization of *ε*-caprolactone

**Scheme 2:**
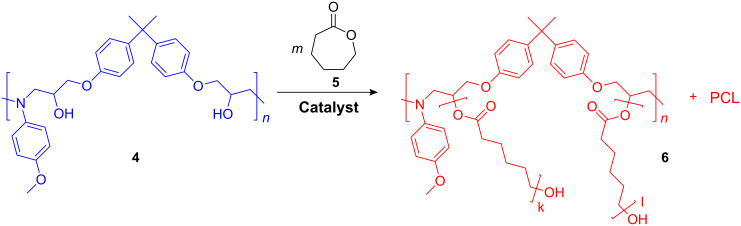
Ring-opening polymerization of *ε*-CL using alcohol units as initiator.

The epoxide-amine adduct **4** was used for preparation of graft copolymer **6** via ring-opening polymerization (ROP) of *ε*-caprolactone (*ε*-CL, **5**), where the secondary hydroxy groups of **4** act as the initiator. This ROP was catalyzed by bismuth(III) trifluoromethanesulfonate, which is known as effective catalyst for ROP [27] (Scheme 2).

The dilution of the hydroxy groups in the obtained product **6** was proven by IR spectroscopy by the nearly complete disappearance of the OH-related absorption band at 3370 cm^−1^. Furthermore, the formation of ester units was confirmed by the appearance of two strong bands at 1721 and 1176 cm^−1^ associated with C=O and C-O stretching vibrations, respectively.

**Figure 5:**
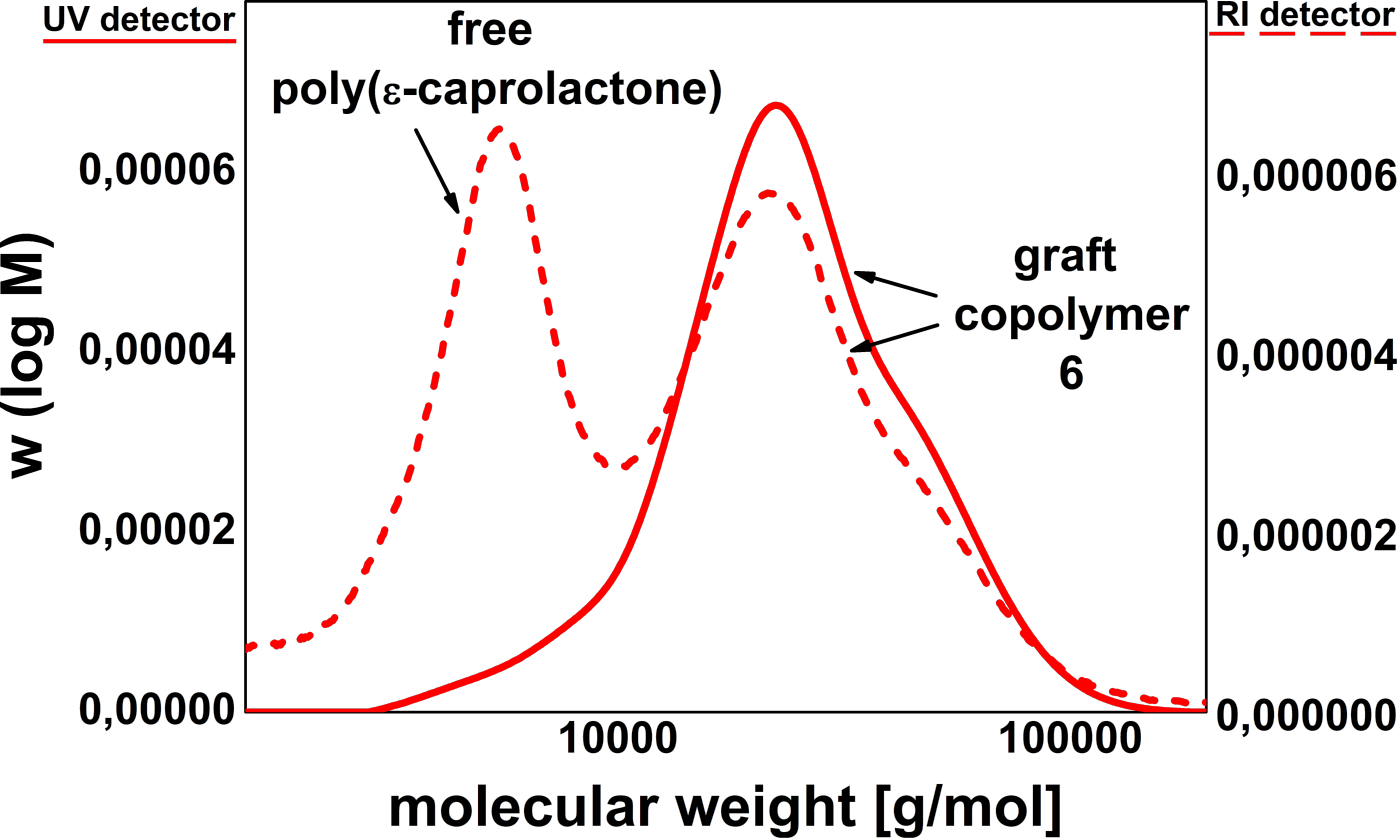
GPC curves of the new formed graft copolymer **6** detected by UV absorption (continuous line) and RI (dashed line).

To prove the existence of grafted polymer **6**, GPC-measurements were carried out. Figure 5 shows the GPC curves of copolymer **6** detected by UV absorption (continuous line) and by RI (dashed line).

The UV absorption of **6** in the GPC diagram (continuous line) clearly indicates the appearance of the aromatic compounds in a higher molecular weight area in comparison with the original polymer **4**. The molecular weights of polymer **6** are about 18000 g mol^−1^ for *M*_n_ and 27000 g mol^−1^ for *M*_w_ with a molecular weight distribution of *M*_w_/*M*_n_ = 1.5. Additionally, the RI curve (dashed line) shows the existence of some non grafted poly(*ε*-caprolactone) (PCL) fractions. PCL is probably formed as a side product since one water molecule in the reaction mixture suffices to start this side reaction.

**Figure 6:**
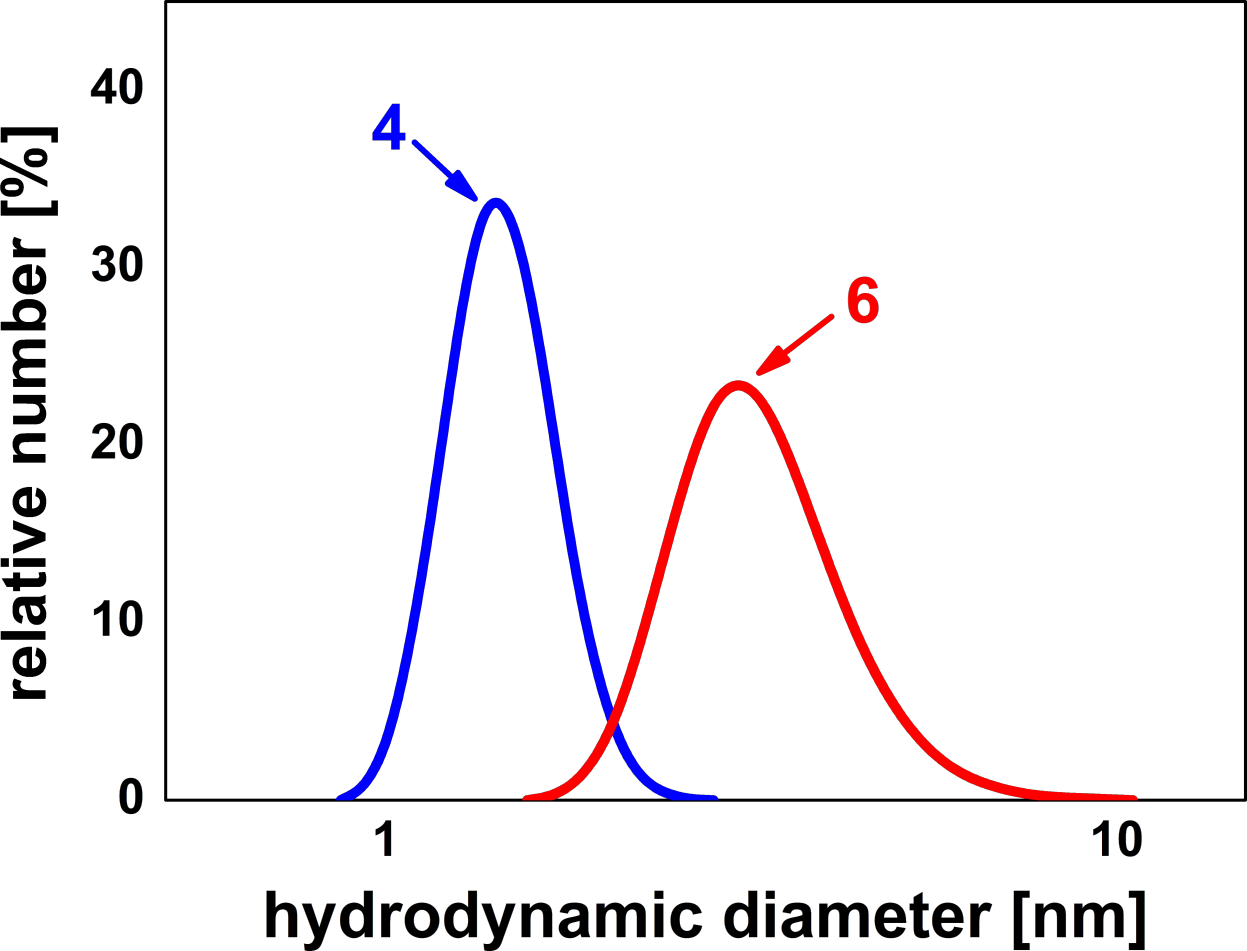
Number-average hydrodynamic diameters of compounds **4** (left) and **6** (right).

The significant increase of molecular weight in comparison with compound **4** was also confirmed by DLS measurements. As shown in Figure 6, the number-average hydrodynamic diameter increases after grafting from 1.45 nm (**4**) to 3.33 nm (**6**).

The DSC curve of grafted compound **6** shows a melting point (*T*_m_) at 50 °C while PCL homopolymer crystallizes at about 55 °C. Obviously the backbone of copolymer **6** reduces the crystalline order.

## Conclusion

A graft copolymer (**6**) was synthesized via ring-opening polymerization of *ε*-caprolactone using an epoxide-amine addition product **4** as an oligomeric initiator. This oligomer can be obtained in a one-pot microwave assisted heterogeneous catalytic transfer hydrogenation, starting from 4-nitroanisole (**2**) in the presence of the diglycidyl ether of bisphenol A (**1**). Product **4** consists of oligomers because of some side reactions. The hydroxy groups of the obtained epoxide-amine adduct **4** were suitable for initiating the ring-opening polymerization of *ε*-caprolactone.

## Experimental

The following commercial products were used: 4-Nitroanisole (97%, Aldrich), 4-methyl-1-cyclohexene (TCI), palladium on activated carbon (Pd/C, 10% Pd, Alfa Aesar) and bismuth(III) trifluoromethanesulfonate (Aldrich) were used without further purification. Diglycidyl ether of bisphenol A (Sigma) was recrystallized from an acetone-methanol mixture (20:80, v/v); mp 44 °C [28]. *ε*-CL was purchased from Aldrich, dried over calcium hydride, distilled under reduced pressure and stored over 0.4 nm molecular sieves under an argon atmosphere. Chloroform-*d* (99.8% d, water <0.01%) was purchased from Euriso-top (France). The solvents obtained were of analytical grade and used as received.

The hydrogenation was performed in a monomodal microwave reactor (CEM Discover S-Class), equipped with an infrared pyrometer for temperature-control and a 300 W power source. Reactions were performed in closed vessels under controlled pressure. Infrared (IR) spectra were recorded on a Nicolet 6700 FT-IR (Fourier transform infrared) spectrometer, equipped with an ATR unit. The measurements were performed in the range of 4000–300 cm^−1^ at room temperature. ^1^H and ^13^C NMR spectra were obtained using a Bruker Avance DRX 500 spectrometer at 20 °C operating at 500.13 MHz for proton and 125.77 MHz for carbon, using deuterated chloroform as solvent. The chemical shift (*δ*-scale) was calibrated to TMS. Elemental analysis (EA) was carried out with a Perkin-Elmer Analyzer 2400 with an accuracy of measurement of ±0.3%. Differential scanning calorimetry (DSC) measurements were performed using a Mettler Toledo DSC 822 controller apparatus equipped with a sample robot TSO801RO. The apparatus was controlled over a temperature range between −40 and 120 °C at a heating rate of 10 K min^−1^. For calibration, standard tin, indium and zinc samples were used. Three heating cycles were conducted. The glass-transition temperature (*T*_g_) values are reported as the average of the second and the third heating cycle using the midpoint method. The melting point (*T*_m_) values are reported as the average peak maxima of the second and the third heating cycle. Matrix-assisted laser desorption-ionization time-of-flight mass spectrometry (MALDI-TOF MS) was performed on a Bruker Ultraflex TOF mass spectrometer. Ions formed with a pulsed nitrogen laser (25 Hz, 337 nm) were accelerated to 25 kV, the molecular masses being recorded in linear mode. Dithranol was used as a matrix and sodium trifluoroacetate (NaTFA) as ionization reagent. The samples were dissolved in THF. Molecular weights and molecular weight distributions were measured by gel permeation chromatography (GPC) using a hydroxyethyl methacrylate (HEMA)-5 *μ*m column set consisting of a precolumn of 4 nm and main colums of 10^3^, 10^2^ and 10 nm. Tetrahydrofuran (THF) was used as the eluent at a flow rate of 1 mL min^−1^. For online detection, a Waters 486 tunable absorbance detector (*λ* = 256 nm) and a Waters 410 differential refractometer were used. The number average molecular weight (*M*_n_), the weight average molecular weight (*M*_w_) and the polydispersity (PD) were calculated by a calibration curve generated by polystyrene standards with a molar mass range from 580 to 1 186 000 Da. Dynamic light scattering (DLS) experiments were implemented on a Malvern HPPS-ET apparatus at a temperature of 25 °C using dimethylformamide (DMF) as solvent. Hellma Suprasil precision cells (110-QS) were used. The particle size distribution was derived from a deconvolution of the measurement number-average autocorrelation function of the sample by the general purpose mode algorithm included in the DTS software. Each experiment was performed five times to obtain statistical information.

### Synthesis of epoxide-amine addition product 4

A 35 mL pressure-resistant microwave test tube provided with a magnetic stirring bar was loaded with diglycidyl ether of bispenol A (**1**) (1.702 g, 5.00 mmol) and 4-nitroanisole (**2**) (0.766 g, 5.00 mmol). The solids were dissolved in 4-methyl-1-cyclohexene (2.404 g, 25.0 mmol). After the addition of 10% Pd/C (125 mg) the tube was sealed with a silicon septum and the mixture irradiated for 5 min at a temperature of 120 °C using a maximum power of 100 W in the monomode microwave reactor. To prevent overheating, the reaction was performed under simultaneous cooling with compressed air (5 psi). After cooling to room temperature, the reaction mixture was dissolved in about 10 mL THF to remove the catalyst by filtration. The solution was concentrated under reduced pressure to give product **4**. The crude product was dissolved in 40 mL THF and precipitated into 400 mL of cold *n*-hexane. The polymer was isolated by filtration and dried under vacuum. A yield of 1.73 g (75%) was obtained.

EA (C_28_H_33_NO_5_)_n_ (463.57)_n_: Calc. C 72.55, H 7.18, N 3.02; Found C 72.40, H 7.26, N 2.92. FT-IR (diamond): 3370 *ν*(OH), 2963, 2930, 2870 *ν*(CH)_CH3, CH2_, 2832 *ν*(CH)_Ar-O-CH3_, 1607, 1582, 1509 *ν*(C=C)_aromatic_, 1462 *δ*(CH), 1362 *δ*_s_(CH), 1296 *δ*(OH), 1237 *ν*(CO)_ArOCH2_ cm^−1^, no absorptions at 1589 *ν*_as_(NO_2_), 1329 *ν*_s_(NO_2_) and 911 *ν*(CH)_epoxide_ cm^−1^. ^13^C NMR (CDCl_3,_ 125 MHz): *δ* (ppm): 155.29 (9), 151.55 (4), 142.58 (1), 141.32 (12), 126.76 (11), 116.13 (3), 113.76 (2), 112.94 (10), 72.21 (C), 69.14 (8’), 68.69 (8), 67.55 (7), 66.99 (7), 65.26 (B), 57.18 (5), 55.50 (6), 54.75 (6), 49.20 (7’), 46.78 (6’’), 43.75 (6’), 40.69 (13), 30.00 (14), 17.75 (A).


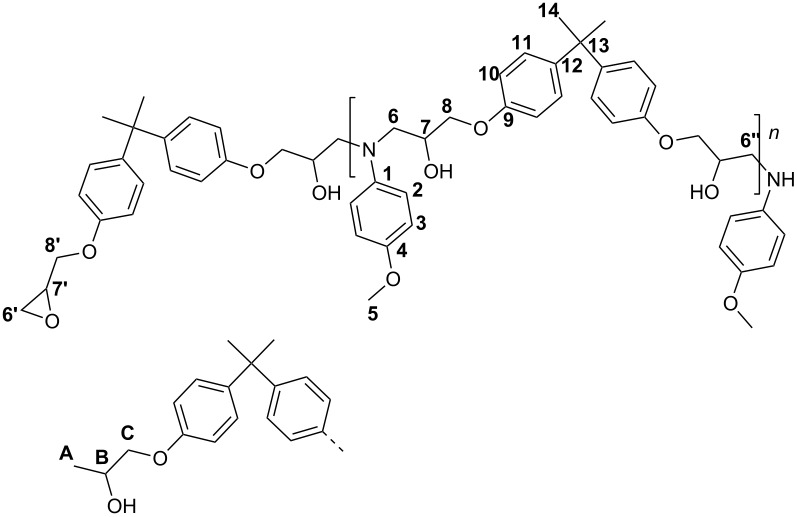


### Synthesis of graft copolymer 6

57.9 mg (0.125 mmol) of the epoxide-amine addition product **4** and 1.142 g (10 mmol) of *ε*-CL (**5**) were mixed with 0.05 mol % of the catalyst bismuth(III) trifluoromethanesulfonate in a 10 mL vial sealed with a septum. The stirred reaction mixture was kept at 100 °C for 4 days. After cooling to room temperature, the reaction mixture was dissolved in 5 mL chloroform and precipitated into 150 mL of cold methanol. The polymer was isolated by filtration and dried under vacuum. A yield of 0.83 g was obtained.

IR (diamond): 2944, 2895, 2865 *ν*(CH)_CH3, CH2_, 1721 *ν*(CO), 1512 *ν*(C=C)_aromatic_, 1471 *δ*(CH), 1365 *δ*_s_(CH), 1293 *δ*(OH), 1239 *ν*(CO)_ArOCH2_, 1176 *ν*(CO) cm^−1^.
